# Sarcoscore: A Novel Approach for Assessing Sarcopenia and Functional Disability in Older Adults

**DOI:** 10.3390/jcm9030692

**Published:** 2020-03-04

**Authors:** Yosuke Osuka, Hunkyung Kim, Hisashi Kawai, Yu Taniguchi, Yuri Yokoyama, Satoshi Seino, Shuichi Obuchi, Akihiko Kitamura, Shoji Shinkai

**Affiliations:** 1 Research Team for Promoting Independence and Mental Health, Tokyo Metropolitan Institute of Gerontology, Tokyo 173-0015, Japan; kimhk@tmig.or.jp; 2 Research Team for Human Care, Tokyo Metropolitan Institute of Gerontology, Tokyo 173-0015, Japan; hkawai@tmig.or.jp (H.K.); obuchipc@tmig.or.jp (S.O.); 3 Japan Environment and Children’s Study Programme Office, National Institute for Environmental Studies, Ibaraki 305-8506, Japan; taniguchi.yu@nies.go.jp; 4 Research Team for Social Participation and Community Health, Tokyo Metropolitan Institute of Gerontology, Tokyo 173-0015, Japan; yokoyama@tmig.or.jp (Y.Y.); seino@tmig.or.jp (S.S.); kitamura@tmig.or.jp (A.K.); 5 Research on Social and Human Sciences, Tokyo Metropolitan Institute of Gerontology, Tokyo 173-0015, Japan; sshinkai@tmig.or.jp

**Keywords:** functional disability, sarcoscore, instrumental activities of daily living, basic activities of daily living

## Abstract

Sarcopenia is associated with instrumental activities of daily living (IADL) and basic activities of daily living (BADL) disabilities. We developed an index for assessing sarcopenia degree (sarcoscore) and compared it to the Asian Working Group for Sarcopenia (AWGS) criteria. Principal component analyses of walking speed, handgrip strength, and skeletal muscle index were performed to develop a sarcoscore using 3088 Japanese population-based cross-sectional data. During the nine-year follow-up, 278 of 2571 and 88 of 2341 participants developed IADL and BADL disabilities, respectively. Adjusted Cox proportional hazards regression models showed that the sarcoscore criteria, defined as proportional to the sarcopenia prevalence diagnosed by the AWGS criteria, had higher hazard ratios (HRs) and 95% confidence interval (CI) for disability onset than the AWGS criteria (IADL disability: 2.19 (1.64–2.93) vs. 1.79 (1.32–2.43), BADL disability: 4.28 (2.63–6.96) vs. 3.22 (1.97–5.27)). The adjusted HRs for IADL and BADL disabilities were reduced by 4% and 8% per point increase in the sarcoscore, respectively. The sarcoscore assessed the degree of sarcopenia and had a satisfactory performance for predicting functional disabilities in older Japanese adults, suggesting its usefulness as a complementary composite marker for clinical diagnosis.

## 1. Introduction

Sarcopenia is defined as a muscle disorder that is characterized by the progressive and generalized loss of skeletal muscle mass or quality, strength, and physical performance [1]. Over the past decades, numerous factors, including decreased anabolic hormone, neuronal motor units, vascular supply, increased systemic inflammation and oxidative stress, and a sedentary lifestyle with malnutrition, have been found to be associated with muscle mass loss and functional decline with aging [2]. Longitudinal epidemiological studies have shown that sarcopenia is associated with adverse outcomes, such as an increased incidence of falls [3,4], disability in instrumental activities of daily living (IADL) or basic activities of daily living (BADL) [5,6,7], hospitalization [6,8], institutionalization [5], and mortality [5,6,9]. Therefore, it is very important to assess sarcopenia in the older population in a clinical setting.

In 1998, Baumgartner et al. [10] proposed an operational definition of sarcopenia by measuring appendicular lean mass (ALM) using dual-energy X-ray absorptiometry (DXA). They defined sarcopenia as having a skeletal muscle index (SMI) (calculated by dividing ALM (kg) per body height (m) squared) ≥2 standard deviations below the mean of the reference group in a young population. Subsequently, muscle strength measurements, including handgrip strength and chair stand tests, were included in the operational definition of sarcopenia before measurements of muscle mass [1], as it has been recognized that muscle strength is better than muscle mass as a predictor of adverse outcomes [11,12]. Also, physical performance measures, including walking speed, short physical performance battery, timed up-and-go test, and 400-m walk test, were used in the operational definition to identify the severity of sarcopenia [1], as these functional parameters predict adverse outcomes [13,14]. Recently, interest regarding the operational definition of sarcopenia has been increasing worldwide. Although no global consensus regarding the operational definition of sarcopenia has yet been reached, at least six major operational definitions have been proposed by international working groups or national projects [1,15,16,17,18,19]. The European Working Group on Sarcopenia in Older People (EWGSOP) first proposed the operational criteria for sarcopenia by measuring muscle strength, muscle mass, and physical performance parameters, and this measure became the most widely used globally [20]. Four of the six major operational definitions use these three parameters [1,16,18,19]. In Asia, the Asian Working Group for Sarcopenia (AWGS) decided to take similar approaches as EWGSOP for the diagnosis of sarcopenia; however, they used different cut-off values for these measurements in order to suit the Asian population [19].

Sarcopenia diagnosis based on the AWGS had good predictive validity for the incidence of physical limitations and mortality; this was similar to that obtained using the other operational definitions [21]. The AWGS diagnostic algorithm categorizes patients based on cut-off values and provides a clear definition of whether patients have sarcopenia. However, this algorithm is unable to provide a detailed information about the degree of change of sarcopenia over time, and how closely their condition resembles sarcopenia, because it measures sarcopenia on a categorical scale. Thus, a comprehensive and continuous index that could provide a measure of total health status regarding skeletal muscle mass and function would be useful in clinical settings as “a complementary composite marker” when clinicians diagnose sarcopenia using the AWGS criteria. The development of such a marker could contribute toward a quantitative comprehension of sarcopenia among physicians and patients by representing sarcopenia on a continuous scale. Additionally, it could help physicians and patients in estimating the degree of progression, and the effects of prevention and treatment of sarcopenia. However, to the best of our knowledge, no such marker has previously been developed.

In order to meet this need, we aimed to develop a novel index which can estimate the degree of sarcopenia (sarcoscore) by using integrated data from three population-based samples, and to compare its predictive value to the AWGS criteria in predicting functional disabilities in community-dwelling, older Japanese adults. We hypothesized that sarcoscore would have predictive validity of functional disabilities, as with sarcopenia diagnosis based on AWGS criteria.

## 2. Methods

### 2.1. Study Design

In order to develop the sarcoscore, we conducted a cross-sectional analysis, and in order to examine the association between the index and the onset of functional disability, we conducted a longitudinal analysis.

### 2.2. Setting and Participants

Three population-based cohort samples were subjected to cross-sectional and longitudinal analyses, including participants from the Itabashi Cohort Study, the Kusatsu Longitudinal Study, and the Hatoyama Cohort Study.

#### 2.2.1. Itabashi Cohort Study

Itabashi ward is a large urban area located in the northwest area of 23 special wards in Tokyo, Japan. This study was initiated from 2011 as an open cohort and new participants were added from 2013. The detailed selection process in 2011 has been described in previous publications [22,23]. Briefly, we invited all 6699 residents aged 65–84 years who lived in nine residential areas, who were not living in a nursing home, or did not have overlapping participation from other cohort studies. Those residents participated in this study in 2011 and 2012. New participants aged 65 years of age were added to the cohort in 2013, 2014, and 2017. In 2016, residents who were 65 and 66 years of age were also added. We excluded data of the 2011 survey because a different body composition analyzer was used in that survey. A total of 1537 individuals were recruited and participated in the baseline survey from 2012 to 2017. Those who participated in the baseline survey from 2012 to 2016 were followed-up every year until 2017.

#### 2.2.2. Kusatsu Longitudinal Study

Kusatsu town is a rural community located in the northwest Gunma prefecture in Japan. The Kusatsu Longitudinal Study was an open cohort which was initiated from 2002 and new participants were added every year. The detailed selection process for this cohort was described in a previous study [24]. Briefly, this cohort targeted all residents who used the National Health Insurance aged 65–74 years or the Medical Insurance system for the Elderly aged 75 years and older. New participants aged ≥65 years were added every year. We used data from the surveys conducted from 2008 onwards. In total, 1411 individuals were recruited and participated in the baseline survey from 2008 to 2017. Those who participated in the baseline survey from 2008 to 2016 were followed-up every year until 2017.

#### 2.2.3. Hatoyama Cohort Study

Hatoyama town is a suburban area outside of Tokyo located in the center of Saitama prefecture in Japan. The Hatoyama Cohort Study was initiated from 2010 as a closed cohort. The detailed selection process was described in a previous study [25]. Briefly, 2697 residents aged 65–84 years were stratified by age and residential area and were randomly selected from the basic resident register. In total, 742 individuals were recruited and participated in the baseline survey in 2010. Those participants were followed-up every year until 2017.

**Figure 1:**
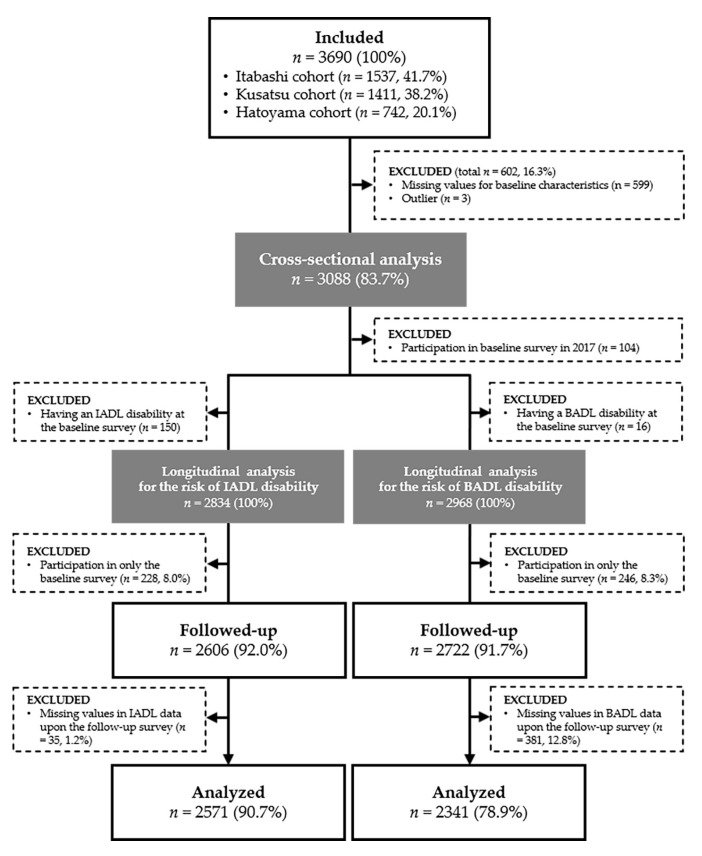
Flowchart of study participants. BADL, basic activities of daily living; IADL, instrumental activities of daily living.

Overall from 2008 to 2017, 3690 adults aged ≥ 65 years participated in the baseline survey; Figure 1 shows the study consort diagram. Of these 3690 older adults, 602 (16.3%) were excluded because they had missing or outlier values for their baseline characteristics. Cross-sectional data of 3088 (women: *n* = 1665, 53.9%) were utilized to develop the sarcoscore formula and for the comparison of baseline characteristics according to the levels of the sarcoscore. In the longitudinal data, the data of 2834 IADL and 2968 BADL participants with initially nondisabled status were followed-up to examine the association between the baseline sarcoscore and the onset of IADL and BADL disabilities. Of the included patients, 2571 (90.7%) IADL and 2341 (78.9%) BADL participants did not have missing data and thus they were able to be followed-up for the whole study period. Thus, they were included in the final multivariable analyses.

All cohort studies were conducted in accordance with the Declaration of Helsinki and the study protocol and all participants provided written informed consent. This study was approved by the Ethics Committee of the Tokyo Metropolitan Institute of Gerontology.

### 2.3. Measurements

#### 2.3.1. Sarcoscore

The variables for developing the sarcoscore were selected from three reliable and validated measurements (walking speed, handgrip strength and SMI) that were used as diagnostic markers for sarcopenia based on six major operational definitions [1,15,16,17,18,19]. All measurements were performed by experienced staff. The measurement methods are described below.

#### 2.3.2. Walking Speed (WS)

WS was measured as the time taken to walk 5 m or 10 m with acceleration and deceleration phases of 3 m each. The examiner instructed the participant: “Please walk on this pathway at your usual speed”. After the participant stood with their feet touching a start-point marked at 0 m, the examiner said “Are you ready? Please, go ahead”. As the participant began to walk, the examiner pushed start button of the stopwatch on the marked at 3 m and pushed stop button of the stopwatch on the marked at 8 m (or 13 m) [26]. As the participant stepped on the mark at 11 m (or 16 m), the examiner recorded time taken to walk the distance. We calculated WS by dividing the distance (5 m or 10 m) by the time taken. The measurements were performed in a single trial. The intra-class correlation coefficients (ICCs) and 95% confidence intervals (95% CIs) of shorter (4 m) and longer (10 m) walk tests were 0.98 (0.96–0.99) and 0.96 (0.94–0.98), respectively, and the standard errors of the mean measurement (SEM) were 0.005 m/s and 0.008 m/s, respectively [27].

#### 2.3.3. Handgrip Strength (HGS)

HGS was measured using a Smedley-type dynamometer [26]. First, the examiner instructed the participant with a demonstration and stated: “Please hold this device like this and squeeze as hard as you can.” The participant stood naturally and gripped the device with their dominant hand at their side. The examiner then said “Are you ready? Please squeeze as hard as you can.” As the participant began to squeeze, the examiner said, “Harder! Harder! Harder! …Relax” [28]. HGS was measured twice in the Kusatsu Longitudinal Study and the Hatoyama Cohort Study and once in the Itabashi Cohort Study. In the studies that took two measurements, the best results were used in analyses. When the best values of the 2 measurements were used, the ICC (95% CI) and SEM (kg) of HGS were 0.94 (0.91–0.96) and 1.88 kg, respectively [29].

#### 2.3.4. Skeletal Muscle Index (SMI)

ALM was measured using a bioelectrical impedance analyzer (Inbody 720, Biospace Co. Ltd., Seoul, Korea). This analyzer consists of a tetrapolar, eight-point tactile electrode system that separately measures impedance of the trunk, arms and legs by using six different frequencies (1, 5, 50, 250, 500, and 1000 kHz) and has a good validity for estimating ALM in a community-dwelling older population, compared to estimation using the standard dual-energy X-ray absorptiometry method [30]. Participants were instructed to wipe the bottom of their feet and hands with a wet tissue. Then, examiner instructed to participants to stand on the analyzer and grip the electrical handrails. The participants were instructed to fully extend their arms and to lift them from their side at a 20-degree angle. The examiner instructed the participant not to talk or move during the measurement. The analyzer automatically computed the lean mass of the arms and legs. We calculated ALM by summing the parameters. ALM was converted to SMI standardized by body height squared (kg/m^2^) [31].

### 2.4. Primary Outcome Measures

The primary outcome was onset of disability in functional capacity. Functional capacity in older adults consists of two abilities: IADL and BADL [32]. IADL involve slightly complex physical activities using instruments and vehicles, including cooking, cleaning, using public transportation, and taking medicines [32]. Conversely, BADL involve more basic physical activities including eating, dressing, and going to the toilet [33]. IADL was measured using a subscale of the instrumental self-maintenance of the Tokyo Metropolitan Institute of Gerontology index of competence (TMIG-IC) [34,35,36]. Participants were asked about ability of performing IADL using five questions and answered either “yes: scored as 1 point” or “no: scored as 0 point”. We summed the five items for a score of 0 to 5 points, with a higher score indicating better IADL ability. Onset of IADL disability was defined as a decrease ≥1 point in follow-up surveys from 5 points (full marks) in the baseline survey [37,38]. BADL was assessed using a modified form of the Katz index [33,37]. We asked participants about ability of performing BADL using five questions and asked participants to select from three choices (intact, scored as 1 point; partially dependent, scored as 0 points; completely dependent, scored as 0 points). The five items were summed for a score ranging from 0 to 5 points, with a higher score indicating better BADL ability. We defined the onset of BADL disability as a decrease ≥1 point in the follow-up surveys from 5 points (full marks) in the baseline survey [38,39].

### 2.5. Other Covariates

Age, sex, height, weight, body mass index (BMI), educational attainment, hospitalization, medical histories, and cognitive function were measured as other covariates. Educational attainment was classified into ≥9 years (graduation from junior high school or higher) or <9 years. We asked participants whether they experienced hospitalization over the past year. Additionally, participants were asked about their medical history including stroke, heart disease, hypertension, hyperlipidemia, diabetes, and chronic obstructive pulmonary disease (COPD) over the past year. Cognitive function was measured using the Mini-Mental State Examination. We defined cognitive impairment as <24 points [40].

### 2.6. Statistical Analysis

First, we applied principal component analysis to the three variables of WS, HGS, and SMI, in order to develop the sarcoscore. Each standard score of the three measurements was multiplied to the first principal component score coefficient which was computed automatically by principal component analysis. Principal component analysis was stratified by sex. We then summed these variables and defined it as the sarcoscore that represents the first principal component score. To help users (physicians and patients) understand the significance of the sarcoscore per point, the sarcoscore was calculated as the T score that described a normal distribution, with a higher score indicating better muscle condition. Next, to identify the optimal cut-off values of the sarcoscore that could discriminate sarcopenia diagnosed using the AWGS criteria [19], we conducted receiver operating characteristic (ROC) analyses. The area under the curve (AUC) was determined by plotting the sensitivity against (1 − specificity) of the sarcoscore, and more than 0.9 can be highly accurate. The cut-off values were selected using the Youden index. Analysis of variance for continuous variables or logistic regression analysis for categorical variables were applied to test trends between the baseline characteristics and the terciles of the sarcoscore. Subsequently, we classified participants into low and high sarcoscore groups so that it was proportional to the sarcopenia prevalence diagnosed by AWGS criteria and defined this classification as the sarcoscore criteria. The cumulative hazard for the functional disabilities until the end of the follow-up period based on the sarcoscore criteria, was calculated using the Kaplan-Meier method. Individuals who were lost-to-follow-up were censored at the last follow-up year. Significant differences in the cumulative hazard curves between the sarcoscore criteria were evaluated using log-rank tests. Finally, Cox proportional hazards models, adjusted for age, sex, cohort area, educational level, hospitalization, stroke, heart disease, diabetes, and cognitive impairment were used to estimate the hazard ratio of the AWGS and sarcoscore criteria for the onset of functional disabilities. The adjusting variables were selected because they were known [5,6,7] or suspected risk factors for functional disability, and were potential confounders. The heterogeneity in the relationship between subgroups were tested by adding multiplicative interaction terms (risk factors × sarcoscore) to the corresponding Cox models. All analyses were performed using IBM SPSS version 25.0 (IBM Corp., Armonk, New York, USA), with *p* values of <0.05 considered to be significant.

## 3. Results

### 3.1. Development of Sarcoscore by using Principal Component Analysis

A principal component analysis extracted the first principal component with corresponding eigenvalue ≥1.0 in both men and women. These components accounted 53.3% and 53.0% of the total variance in men and women, respectively. First principal component score coefficients were obtained as follows: HGS: 0.838 in men, 0.835 in women; SMI: 0.740 in men, 0.694 in women; WS: 0.591 in men, 0.642 in women. We multiplied these coefficients to standard scores for the three measurements and developed the sarcoscore formula by summing those values. Finally, these formulas were converted to T scores as follows.

(1) $${Sarcoscore}_{for\;men}=10\times \left[\left(0.838\times \frac{X_{1}-34.20}{7.03}\right)+\left(0.740\times \frac{X_{2}-7.36}{0.77}\right)+\left(0.591\times \frac{X_{3}-1.35}{0.24}\right)\right]+50$$

(2) $${Sarcoscore}_{for\;women}=10\times \left[\left(0.835\times \frac{X_{1}-21.14}{4.90}\right)+\left(0.694\times \frac{X_{2}-5.88}{0.67}\right)+\left(0.642\times \frac{X_{3}-1.34}{0.25}\right)\right]+50$$

$$X_{1}=HGS\;\left(kg\right),\;X_{2}=SMI\;\left(kg/m^{2}\right),\;X_{3}=WS\;\left(m/s\right)$$

### 3.2. Cut-off Values of Sarcoscore for Discriminating Sarcopenia Diagnosed by AWGS Criteria

**Figure 2:**
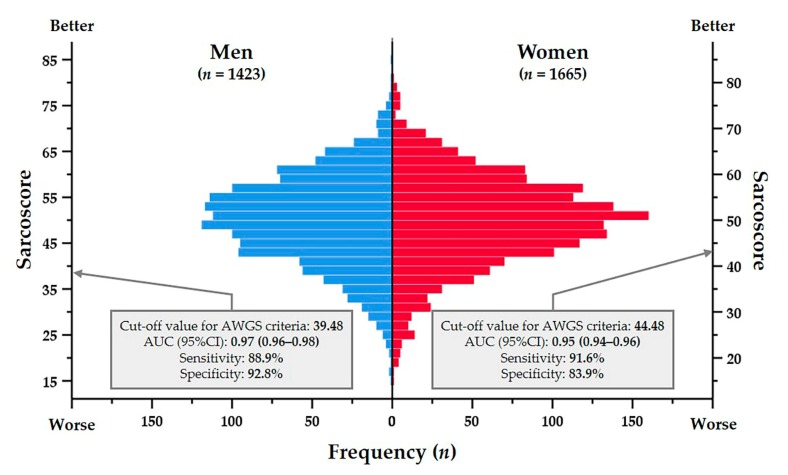
Distribution of sarcoscore on study population and its cut-off values for sarcopenia diagnosed by the Asian Working Group for Sarcopenia criteria. Note: AUC, area under the curve; AWGS, Asian Working Group for Sarcopenia; CI, confidence interval.

Figure 2 shows the distribution of sarcoscore on this population and its cut-off values for discriminating sarcopenia diagnosed by the AWGS criteria. The AUCs and 95% CIs of sarcoscore for discriminating sarcopenia were 0.97 (0.96–0.98) for men and 0.95 (0.94–0.96) for women. The cut-off values of sarcoscore for discriminating sarcopenia were 39.48 (sensitivity: 88.9%, specificity: 92.8%) for men, and 44.48 (sensitivity: 91.6%, specificity: 83.9%) for women.

### 3.3. Sarcoscore and Baseline Characteristics

Table 1 shows the cross-sectional associations between the baseline characteristics and the terciles of the sarcoscore. Trend analyses indicated that higher sarcoscores were significantly associated with younger age, higher educational levels, and lower prevalence of hospitalization, stroke, heart disease, diabetes, and cognitive impairment.

### 3.4. Sarcoscore and the Risk of Functional Disabilities

**Figure 3:**
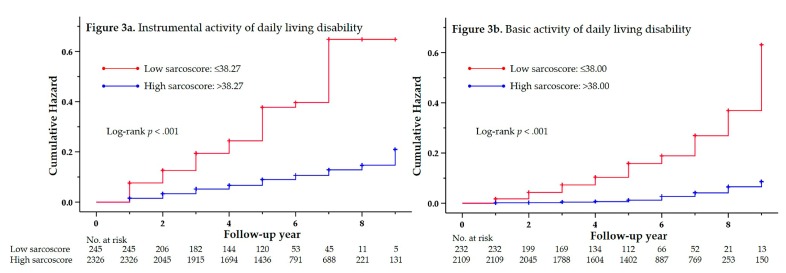
Cumulative risk of disabilities in instrumental and basic activities of daily living according to the sarcoscore criteria. The sarcoscore criteria was set so that the sarcopenia prevalence would be similar to that of the sarcopenia prevalence based on the AWGS criteria. BADL, basic activities of daily living; IADL, instrumental activities of daily living.

IADL and BADL disabilities were observed in 278 participants (10.8%) during the follow-up period of 13,122 person-years (median of the follow-up: five years) and in 88 participants (3.8%) during the follow-up of 11,846 person-years (median follow-up: five years), respectively. Figure 3a,b shows the number of participants followed-up for nine years and the results of the cumulative hazard based on the sarcoscore criteria plotted using the Kaplan–Meier method. There were significant differences in the cumulative hazard curves for the functional disabilities between two groups (*p* for log-rank test, <0.001).

Table 2 shows the results of the crude and adjusted Cox proportional hazards models in predicting the hazard ratios for IADL and BADL disabilities based on the AWGS and sarcoscore criteria. Participants with sarcopenia or a low sarcoscore in the baseline survey were significantly more likely to experience BADL and IADL disabilities during the follow-up period compared to participants without sarcopenia and those with a high sarcoscore. Although both sarcopenia and low sarcoscore groups in the baseline survey had significantly higher rates for IADL and BADL disabilities during the follow-up period, even after adjustment for potential confounders, the sarcoscore criteria had a higher hazard ratio (HR) for the onset of the disabilities than the AWGS criteria (IADL disability, AWGS criteria: HR: 1.79 (95% CI: 1.32–2.43) vs. sarcoscore criteria: HR: 2.19 (95% CI: 1.64–2.93); BADL disability, AWGS criteria: HR: 3.22 (95% CI: 1.97–5.27) vs. sarcoscore criteria: HR: 4.28 (95% CI: 2.63–6.96). When the sarcoscore were included in the model as continuous variables, the adjusted hazard ratios for IADL and BADL disabilities were significantly reduced by approximately 4% and 8% per point increase in the sarcoscore, respectively.

**Figure 4:**
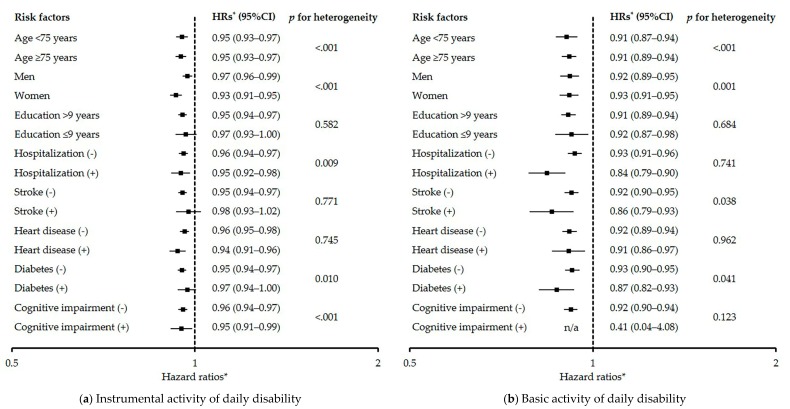
The influence of sarcoscore (for +1 point) on the risk of disabilities of instrumental activities of daily living and basic activities of daily living according to the subgroup of potential risk factors. Squares indicate point estimate of adjusted hazard ratio, and bars indicate 95% confidence intervals. *: Adjusted for age, sex, cohort area, low education level, hospitalization, stroke, heart disease, diabetes, and cognitive impairment. n/a: not applicable. The variables relevant to the subgroup were excluded from the corresponding model.

In the stratified analyses based on potential risk factors, significant differences in heterogeneity were found in subgroups of age, sex, hospitalization, diabetes, and cognitive impairment for IADL disability (Figure 4a), and age, sex, stroke, and diabetes for BADL disability (Figure 4b).

## 4. Discussion

The present study has two novelties. First, to the best of our knowledge, the present study was the first to develop a comprehensive and continuous index (sarcoscore) for assessing sarcopenia, which summarized three-dimensional information including HGS, SMI, and WS into a one-dimensional format using three population-based samples. Second, this study was the first to identify that the sarcoscore had enough predictive value in predicting functional disabilities as compared to the AWGS criteria. These results supported our hypothesis and suggested that the sarcoscore would be useful in assessing the degree of sarcopenia and predicting functional disabilities in community-dwelling older Japanese adults. Thus, it may help physicians and patients in estimating the degree of progression, prevention, and treatment effect of sarcopenia and may contribute toward a quantitative understanding of those process.

The cut-off value for discriminating sarcopenia was extremely accurate (Figure 2), since the sarcoscore was algorithmized using sarcopenia diagnostic markers. Physicians and patients can visually confirm the current relative status of skeletal muscle health by comparing the patients’ values to those cut-off values. Additionally, 50 points of sarcoscore shows the average value of sarcoscore of this population, thus facilitating comparison with the general Japanese older adult population.

Previous studies have reported that individual sarcopenia diagnostic markers are associated with the risk of adverse events in the elderly population [7,41,42]. HGS, WS, and SMI are associated with blood biomarkers including inflammatory and hormone markers in the older population [43,44,45,46,47]. These biomarkers can explain the physiological background of sarcopenia and physical frailty, but they are only able to capture single aspects of the condition and are weakly associated with clinically meaningful adverse outcomes [48]. Thus, it is very important to assess “functional markers” such as HGS and WS in the clinical setting. Our results support those from previous studies and reveal that not only individual functional markers, but also a composite functional marker have predictive validity for the onset of functional disabilities. These findings are robust since the significance of the results remained unchanged even after adjusting for some covariates. Additionally, the sarcoscore had slightly higher predictive value in the prediction of functional disabilities than the AWGS criteria (Table 2). This result indicates that an integration assessment using a summarized value would have a larger amount of information for predicting functional disabilities than a stepwise assessment using cut-off values. In turn, this suggests that the sarcoscore had enough predictive validity compared to the AWGS criteria. The AWGS criteria were assessed by using the cut-off values of HGS, WS, and SMI to classify patients, and such algorithms may exclude non-negligible information. For example, a patient who meets the cut-off value of HGS, but not WS and SMI, would not be diagnosed as having sarcopenia when a physician assesses the patient using the AWGS algorithm [19]. However, it cannot be concluded that this patient had no risk of functional disabilities. The sarcoscore could play an important role in comprehensive assessment of the prognosis of patients in such unclear clinical situations, as serve as a complementary composite marker when using the AWGS criteria.

A sarcoscore would allow for the largest clinical benefit in terms of allowing continuous assessment of the degree of progress with aging and improvement due to treatments. For example, in the case of an older man for whom all measurements were average upon the baseline survey, if all measurements worsened by 1% during the 1-year followed-up period, the sarcoscore would decrease by 1.4 points. Theoretically, the degree of this reduction would increase the risk of IADL disability by 5.6%. Thus, the sarcoscore can acutely respond to the slight change in all of the sarcopenia-related markers with aging and can predict adverse outcomes. Conversely, in the case of an older woman for whom all measurements were average upon the baseline survey, if all measurements were improved by 3% based on a treatment, the sarcoscore would also increase by 3.9 points. This gain would sequentially decrease the risk of BADL disability by 31.2%. When physicians and researchers assess the effects of intervention programs using the algorithm of cut-off values, then the effects of the program can only be assessed as a reversal rate; however, if the sarcoscore is used, the degree of the effects can be assessed using a continuous variable. Physicians and researchers should be aware that risk estimates per point of sarcoscore differ depending on the potential risk factors (Figure 4). If the assessments based on the sarcoscore are visualized using various applications in the future, this will contribute toward motivating patients to engage in the prevention of, and seek treatment for sarcopenia.

Our study has several strengths in terms of its methodology. First, the sarcoscore might well reflect a population of elderly, community-dwelling Japanese adults, since present study integrated three population-based cohorts from the urban (Itabashi), suburban (Hatoyama), and rural (Kusatsu) areas in Japan. This increases the generalizability of the use of the sarcoscore among various community settings in Japan. Second, the sample size in the present study was relatively large compared to that of previous population-based studies which examined the associations between sarcopenia-related markers and functional disabilities in Australia [5], Italy [7], and Germany [49]. This minimizes the risk of *β* error and enables clearer identification of the strength of the association between the sarcoscore and the onset of functional disabilities in this population.

Conversely, our study was also subject to several limitations. First, unmeasured information including depression symptoms, smoking and drinking status, and habitual dietary and physical activity levels are potential confounders that were not accounted for in this study. Therefore, the strength of the association between the sarcoscore and the risk of functional disabilities, which was adjusted for other potential confounders, is unclear. Future studies should be designed to collect these data prospectively. Second, the use of the sarcoscore should be limited to older Japanese adults, since the sarcoscore formula would change depending on the mean and standard deviation of the sample. Also, associations of sarcoscore with other adverse outcomes were not examined. In the future, it would be useful to develop a sarcoscore for use in different populations and to assess whether the sarcoscore is associated with other adverse outcomes.

## 5. Conclusions

The present study developed a sarcoscore to assess the degree of sarcopenia and identified that it had sufficient predictive value in predicting the functional disabilities in community-dwelling older Japanese adults compared to the AWGS criteria. These results suggest that a sarcoscore may contribute to assisting physicians and patients in the estimation of the degree of progression and the effects of the treatment of sarcopenia in clinical settings, as “a complementary composite marker” when using AWGS criteria.

## Figures and Tables

**Table 1:** Comparison of baseline characteristics according to the tercile of the sarcoscore.

	All	Tercile of the Sarcoscore			
		Low	Middle	High	*p* for Trend
	*n* = 3088	*n* = 1030	*n* = 1028	*n* = 1030	
Age, years	71.4 (5.8)	74.6 (6.2)	70.9 (5.2)	68.6 (4.1)	<0.001
Sex, women	1665 (53.9)	548 (53.2)	570 (55.4)	547 (53.1)	0.965
Height, cm	156.6 (8.8)	153.5 (8.7)	156.7 (8.2)	159.7 (8.4)	<0.001
Weight, kg	56.8 (10.6)	52.0 (9.2)	56.4 (9.3)	62.1 (10.6)	<0.001
Body mass index, kg/cm^2^	23.1 (3.2)	22.0 (3.0)	22.9 (2.8)	24.3 (3.4)	<0.001
Education, <9 years	193 (6.3)	136 (13.2)	36 (3.5)	21 (2.0)	<0.001
Hospitalization, yes	269 (8.7)	127 (12.3)	71 (6.9)	71 (6.9)	<0.001
Medical history					
Stroke, yes	187 (6.1)	80 (7.8)	62 (6.0)	45 (4.4)	0.001
Heart disease, yes	441 (14.3)	169 (16.4)	152 (14.8)	120 (11.7)	0.002
Hypertension, yes	1316 (42.6)	469 (45.5)	417 (40.6)	430 (41.7)	0.082
Hyperlipidemia, yes	965 (31.3)	320 (31.1)	325 (31.6)	320 (31.1)	>0.990
Diabetes, yes	393 (12.7)	155 (15.0)	134 (13.0)	104 (10.1)	0.001
COPD, yes	94 (3.0)	37 (3.6)	30 (2.9)	27 (2.6)	0.201
MMSE, point	28.1 (2.2)	27.5 (2.7)	28.3 (2.1)	28.6 (1.6)	<0.001
Cognitive impairment, yes	120 (3.9)	77 (7.5)	30 (2.9)	13 (1.3)	0.001
IADL disability, yes	152 (4.9)	99 (9.6)	37 (3.6)	16 (1.6)	<0.001
BADL disability, yes	18 (1.5)	15 (1.5)	3 (0.3)	0 (0.0)	<0.001
Sarcoscore, point	50.0 (10.0)	39.1 (6.3)	50.4 (2.3)	60.6 (5.0)	<0.001
Walking speed, m/sec	1.34 (0.20)	1.18 (0.23)	1.36 (0.19)	1.50 (0.21)	<0.001
Handgrip strength, kg	27.2 (8.8)	21.8 (7.1)	27.1 (7.3)	32.6 (8.5)	<0.001
Skeletal muscle index, kg/m^2^	6.56 (1.03)	6.03 (0.90)	6.53 (0.89)	7.12 (0.99)	<0.001

Data are shown as the mean (standard deviation) or *n* (%). COPD, chronic obstructive pulmonary disease; MMSE, Mini-Mental State Examination; IADL, instrumental activities of daily living; BADL, basic activities of daily living.

**Table 2:** Hazard ratios for the onset of instrumental activities of daily living, and basic activities of daily living disabilities according to the Asian Working Group for Sarcopenia and the sarcoscore criteria.

	Events/All	Crude Model		Adjusted Model ^a^	
		HR (95% CI)	*p*	HR (95% CI)	*p*
**IADL Disability**					
AWGS criteria					
Non-sarcopenia	219/2326	Reference		Reference	
Sarcopenia	59/245	2.73 (2.05–3.64)	<0.001	1.79 (1.32–2.43)	<0.001
Sarcoscore criteria ^b^					
High sarcoscore, >38.27	205/2326	Reference		Reference	
Low sarcoscore, ≤38.27	73/245	4.06 (3.11–5.31)	<0.001	2.19 (1.64–2.93)	<0.001
Sarcoscore, for +1 point	278/2571	0.93 (0.92–0.94)	<0.001	0.96 (0.94–0.97)	<0.001
**BADL Disability**					
AWGS criteria					
Non-sarcopenia	58/2109	Reference		Reference	
Sarcopenia	30/232	5.43 (3.49–8.45)	<0.001	3.22 (1.97–5.27)	<0.001
Sarcoscore criteria ^b^					
High sarcoscore, >38.00	53/2109	Reference		Reference	
Low sarcoscore, ≤38.00	35/232	7.95 (5.18–12.19)	<0.001	4.28 (2.63–6.96)	<0.001
Sarcoscore, for +1 point	88/2341	0.90 (0.88–0.91)	<0.001	0.92 (0.90–0.94)	<0.001

IADL, instrumental activities of daily living; BADL, basic activities of daily living; AWGS, Asian Working Group for Sarcopenia; HR, hazard ratio; CI, confidence interval. ^a^ Adjusted for age, sex, cohort area, education, hospitalization, stroke, heart disease, diabetes, and cognitive impairment. ^b^ The sarcoscore cut-point was set so that the sarcopenia prevalence would be similar to that of the sarcopenia prevalence based on the AWGS criteria.
